# Key Parameters to Tailor Hollow Silica Nanospheres for a Type I Porous Liquid Synthesis: Optimized Structure and Accessibility

**DOI:** 10.3390/nano11092307

**Published:** 2021-09-06

**Authors:** Justine Ben Ghozi-Bouvrande, Stéphane Pellet-Rostaing, Sandrine Dourdain

**Affiliations:** ICSM, Univ Montpellier, CEA, CNRS, ENSCM, 30207 Marcoule, France; justine.benghozibouvrande@cea.fr (J.B.G.-B.); stephane.pellet-rostaing@cea.fr (S.P.-R.)

**Keywords:** porous liquids, silica hollow nanospheres, surfactant templating

## Abstract

Based on silica hollow nanospheres grafted with an ionic shell, silica-based type I porous liquids remain poorly exploited, despite their huge versatility. We propose here to explore the main synthesis step of these promising materials with a thorough characterization approach to evaluate their structural and porous properties. Modifying the main synthesis parameter, the mechanism of the spheres’ formation is clarified and shows that the calcination temperature, the surfactant concentration as well as the micelle swelling agent concentration allow tuning not only the size of the nanospheres and internal cavities, but also the silica shell microporosity and, therefore, the accessibility of the internal cavities. This study highlights the key parameters of hollow silica nanospheres, which are at the basis of type I porous liquids synthesis with optimized structural and porous properties.

## 1. Introduction

Porous liquids are an emerging class of liquid materials that contain permanent cavities/pores, the minimum size of which should at least allow a single molecule to be adsorbed. First mentioned in 2007 by O’Reilly et al. [1], porous liquids are decomposed into the following three classes: Type I is constituted of solid hollow cavities that become liquids after being grafted with organic functions. The first example of a Type I porous liquid was proposed in 2015 with subnanometric spherical cavities [2] and a second example was more recently proposed with silicalites [3]. The two other types, II and III, require the addition of a solvent to solubilize the host’s cavities. Type II porous liquids gather molecular hosts cavities that can be organic [4,5,6,7] or inorganic [8], while type III concerns suspensions of microporous frameworks as MOF cages [9] or zeolites [5]. The solvent choice is often critical within these two classes as it must be large enough to not enter the cavities.

In the literature, porous liquids have been mainly proposed for gas capture applications [10]. They exhibit remarkable properties such as fast gas diffusion, high gas solubilities [6,9] and are promising for gas separation thanks to modular selectivities [11]. More recently, they were shown as tailorable and tunable materials for gas transport and sorbent [11,12,13]. However, as porous solid materials that find use in broader applications [14,15,16,17] (drug delivery, energy storage and conversion, catalysis, optical, adsorption, separation, sensing, etc.), porous liquid could be envisaged for much more applications if their synthesis and physico-chemical parameters are mastered.

In this article, we focus on Type I porous liquids, following the synthesis proposed by Zhang et al. in 2012 [2]. This type of porous liquid presents many advantages as it both exploits the versatility of templated mesoporous silica nanoparticles and the property of Nanoscale Ionic Materials (NIMS) to control their state from powdery solids to flowing liquids [18,19,20].

Based on the formation of sol-gel silica nanoparticles templated by surfactant self-assembly, the solid nanoparticles as well as the pores size and shapes are adjustable and functionalizable. In their study, Zhang et al. proposed 15-nanometer spherical hollow nanoparticles with a core radius size of approximately 6 nm. To obtain a porous liquid, they turned these solid nanoparticles into NIMS by grafting their external surface with an ionic corona. While very promising, these materials remain poorly exploited because they are poorly studied and characterized.

In the aim of optimizing the use of such materials for gas separation, or of considering them for other applications, it is of essential interest to characterize them in more detail. We propose here to carefully investigate the synthesis parameters of the hollow silica nanospheres to understand their formation mechanisms in order to optimize the accessibility of the cavities, which is a requisite for numerous potential applications. To this extent, we attempt, in this paper, to evaluate the effect of the main synthesis parameters of these materials on their structural and porous properties with a complete characterization approach. The effect of key parameters such as calcination temperature, surfactant content and swelling agent concentration are evaluated using Transmission Electron Microscopy, Small Angle X-ray Scattering, Infra-Red spectroscopy, ThermoGravimetric Analysis and N_2_ sorption isotherms.

## 2. Materials and Methods

### 2.1. Materials

All chemicals were purchased from commercial sources and used without further purification. Tetramethyl orthosilicate, Pluronic F-127, poly (ethylene glycol) 4-nonylphenyl 3-sulfopropyl ether potassium salt, (3-mercaptopropyl) trimethoxysilane (95%), Mesitylene and acetone were purchased from Sigma-Aldrich (Saint Quentin Fallavier, France); and N,N-didecyl-N-methyl-N-(3-trimethoxysilylpropyl) ammonium chloride (42% in methanol) was purchased from ABCR-Roth Sochiel, France.

### 2.2. Methods

#### 2.2.1. Preparation of Hollow Silica Nanospheres

The hollow silica nanospheres were synthetized by mixing 1.0 g of 1,3,5-trimethylbenzene, 0.87 g K_2_SO_4_ and 1.0 g triblock copolymers F127 in a round flask with 60 mL of deionized water. This solution was stirred between 13 and 16 °C for 4 h. Then, 2.43 g of tetramethoxysilane (TMOS) and 0.78 g of (3-mercaptopropyl)trimethoxysilane (MPTMS) were added to the solution under stirring. After 24 h, the mixture was heated up to 100 °C during 24 h. The solution was then filtered on Büschner and washed 3 times with ultra-pure water. The resulting product was dried at 100 °C and calcinated at 550 °C for 10 h to obtain a white powder of hollow silica nanospheres.

#### 2.2.2. Preparation of Porous Liquid

The hollow silica nanospheres were dispersed in 20 mL of an aqueous solution at pH 8 and sonicated during 10 min. Then, 2.0 mL of organosilane was added to the solution under stirring. After 24 h, the HS grafted was rinsed by centrifugation: 3 times with water and 3 times with EtOH. The white powder was dried at 100 °C overnight. The HS grafted powder was then grafted by adding 15.0 mL of a poly(ethylene glycol) tailed sulfonate solution at 70 °C. After 24 h of mixing, the excess of PEG was extracted by warm toluene (3 times). Ultimately, the aqueous layer was dried in an oil bath at 70 °C for 24 h. The sample was mixed in 15.0 mL of acetone and centrifuged. The clear sol obtained was finally dried in an oil bath at 70 °C overnight.

#### 2.2.3. Characterization

Particles were imaged using Transmission Electron Microscopy (TEM), using a JEOL (Tokyo, Japan) 1400+ at 100 kV equipped with a LaB_6_ filament and a sCMOS JEOL (Tokyo, Japan) Matataki Flash camera. The hollow silica nanospheres or porous liquids were dispersed and mixed with ethanol. A droplet of the solution was dropped off on a carbon grid. The size measurement was performed with ImageJ, on a minimum of 30 spheres (core, shell, total) to have a representative panel of the sample.

Fourier Transform Infrared Spectroscopy (FTIR) was performed on Perkin Elmer (Wellesley, MA, USA) Spectrum 100 spectrometer in transmission mode. A small amount of sample powder was homogeneously dispersed on the beam path. The wavenumber range was from 380 to 4000 cm^−1^. The beam resolution was 0.5 cm^−1^. Each measurement was repeated 4 times.

Thermogravimetric Analysis (TGA) was performed on a Mettler Toledo (Greifensee, Swiss) TG device with the STARe Software V 13.00. Measurements were conducted in alumina’s crucibles of 70 and 150 µL with a significant amount of material (~50 mg). The mass loss was measured between 25 and 1000 °C with a ramp of 5 °C per minute for powders and 2 °C per minute for liquids. After the experiment, the crucible was cooled down under air flux at 30 °C per minute.

Nitrogen adsorption/desorption analysis was carried out with an Micromeritics (Norcross, GA, USA) ASAP-2020 instrument at 77K. Samples were outgassed at 80 °C overnight to reach a pressure below 1 mmHg. The nitrogen gas used for the experiment was 99.8% pure. Surface area and pore diameter were obtained using the Brunauer_Emmett_Teller (BET) method and the Barret_Joyner_Halenda (BJH) model. The micropore volumes were analyzed using the t-plot method with a statistical thickness t obtained from the Harkins and Jura equation.

Small Angle X-ray Scattering (SAXS) experiments were conducted on a home-built device at the ICSM, using a bench built by Xenocs with a molybdenum anode as X-ray source, delivering a high-energy monochromatic beam (wavelength λ = 0.71 Å). The MAR Research 345 detector was located at 760 mm from the sample. The powders were measured, 600 s and the liquids, 3600 s. Calibration into absolute unit was performed using a polyethylene standard. Data treatment was performed with Fit2D.

For small angles, SAXS data were completed with experiments carried out on a homemade instrument at the SWAXS-Lab platform (CEA, Saclay, France). The X-ray source (8 keV energy Genix from Xenocs) produced a collimated beam of 0.8 mm × 0.8 mm on the sample and a flux of around 108 photons/s. The sample-to-detector distance was 114 cm, resulting in a q range of 8 × 10^−3^ to 0.3 Å^−1^ on the detector (Dectris Pilatus 200 K). Calibration of the sample-to-detector distance was obtained with tetradecanol, while direct beam measurement enabled detector counts to be normalized into differential cross section per volume. Acquisition time was 3600 s for each measurement. Data treatment was performed using PYSAXS27. Data modeling was performed with Sasview 5.0.4.

## 3. Results and Discussion

### 3.1. Synthesis and Complete Characterization of Porous Liquids

**Figure 1:**
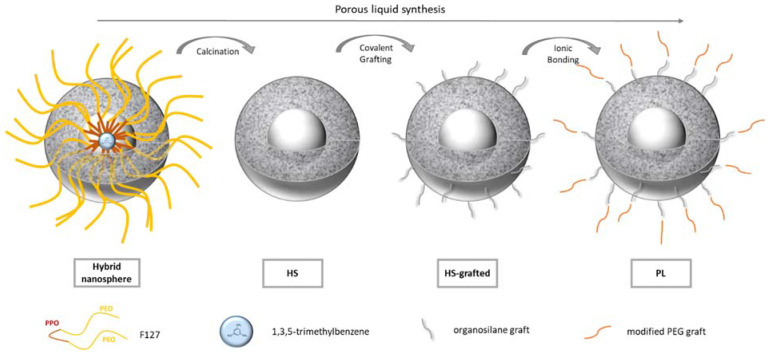
Illustration of the porous liquid synthesis steps.

The Type I porous liquid synthesis proposed by Zhang et al. [2] was reproduced and characterized in detail. As illustrated in Figure 1, porous liquids are obtained following a four-step procedure.

Hollow silica nanospheres are first templated thanks to the self-assembly of pluronic F127 surfactant into spherical micelles. The F127 PPO hydrophobic branches form the core of the micelle, while the PEO hydrophilic parts form the shell. 1,3,5-trimethylbenzene, a hydrophobic component was also added in the solution. It is expected to act as a swelling agent of the hydrophobic core of the micelles. Silica precursors are then added to form a silica corona using a sol-gel process. Being hydrophilic, the silica network polycondenses in the shell of the micelles. At this step, hybrid nanospheres are formed (Figure 1). The surfactant and the oil are then removed by a calcination step to provide hollow nano-spheres (HS in Figure 1). To form the “porous liquid”, an organic corona is finally grafted in two steps at the nanospheres surfaces: first, an organosilane is covalently bonded to the surface (HS grafted in Figure 1); second a modified PEG is linked to the previous organic shell by an ionic bond (PL in Figure 1). This ionic bond is giving the liquid feature to the hollow silica spheres.

**Figure 2:**
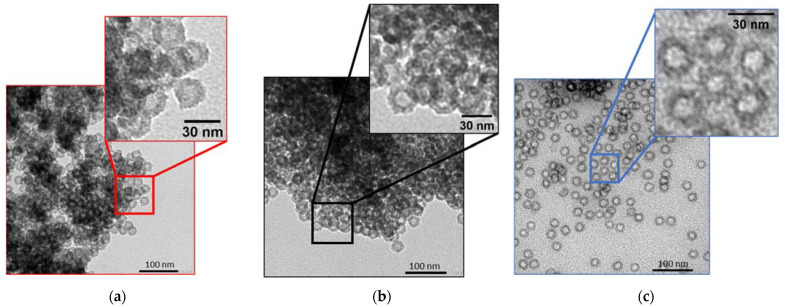
TEM images of (**a**) HS, (**b**) HS grafted and (**c**) PL.

The obtained materials were characterized after each important step of the synthesis: a powder of hollow spheres after calcination (HS), a powder of hollow spheres after organosilane grafting (HS grafted) and a porous liquid (PL). Figure 2 shows the TEM micrographs measured after these three principal synthesis steps. For the three samples, well defined and monodispersed nanospheres, typically of a 30-nanometer total diameter and 15-nanometer core diameter, are observed. It is important to notice that the particles are more widely spaced for the porous liquid. The space between the spheres is associated to the PEG corona grafted at the nanospheres surface. It is also essential to note that the spheres appear empty in the three cases.

**Figure 3:**
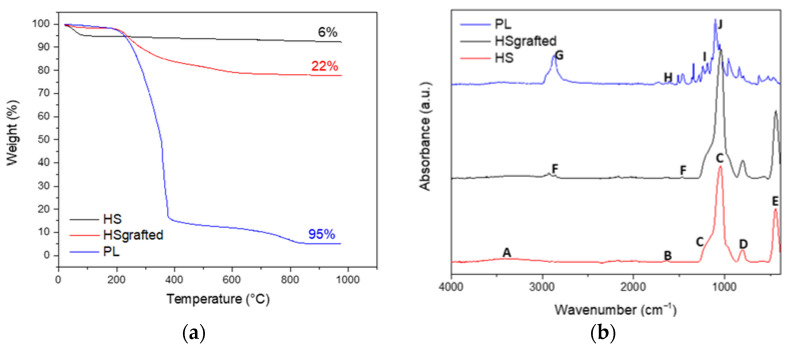
(**a**) TGA curves of HS, HS grafted and PL; (**b**) FTIR spectra of HS, HS grafted and PL.

Thermogravimetric analysis was further applied on the same three samples. Figure 3a shows that the calcinated hollow nanospheres lose about 5 wt% of residual water before 100 °C. Once the spheres are grafted, there is no significant mass loss before 250 °C, for both the HS grafted and the PL samples. As observed by Bourlinos et al. [20], it confirms that the PL is solvent free. A significant loss of mass after 250 °C for the HS grafted and PL samples confirms the presence of the organic corona after the different steps of grafting. It can be estimated that the organic grafts in the HS grafted and in the PL are 22 wt% and 95 wt%, respectively. It is interesting to notice that due to an abundant quantity of modified PEG in its canopy, the final porous liquid contains only 5 wt% of silica.

The composition of the material was also followed by Fourier Transform InfraRed spectroscopy (FTIR) (Figure 3b). For the HS sample, a wide band at 3400 cm^−1^ (A) is assigned to the stretching vibrations of the Si–OH hydroxy groups in the amorphous structure of silica nanospheres. An IR band due to the stretching vibration of H_2_O molecules is also present at 3400 cm^−1^. It is associated with the bending vibration of H_2_O molecules at 1630 cm^−1^ (B) [21]. The broad band at 1050 cm^−1^ (C) and the shoulder at 1170 cm^−1^ (C) are characteristic of the TO and LO modes of the asymmetric stretching vibrations bands of Si–O–Si [21]. The IR band at 800 cm^−1^ (D) can be assigned to the symmetric stretching bands of Si–O–Si, while the band at 480 cm^−1^ (E) is due to the O–Si–O bending vibrations. These characteristic silica IR bands confirm that the silica synthesis using the sol-gel process is efficient. For the HS grafted sample, FTIR also confirms that the organosilane groups are well grafted on the silica. The featured bands of the organosilane grafts corresponding to the stretching and bending vibrations of –CH_2_ backbones are located at 2920, 2840 and 1470 cm^−1^ (F) [2]. Moreover, the intensity ratio of the Si–O–Si bands decreases from 6.28% for HS to 2.74% for HS grafted, suggesting that the increase in the Si–O–Si band is due to the reaction of the Si–OH with the Si–OC of the organosilane graft. The final step to obtain a porous liquid was characterized using FTIR. PEG grafting is confirmed by the presence of characteristic bands at 2874 cm^−1^ (G) for aliphatic compounds, 1651 cm^−1^ (H) for phenyl, 1205 cm^−1^ (I) for sulfonate and 1105 cm^−1^ (J) for ether [2]. As estimated by TGA, the PL sample contains only 5 wt% of silica when the PEG is grafted. It is consistent with the lower intensity of the bands assigned to the silica network.

**Figure 4:**
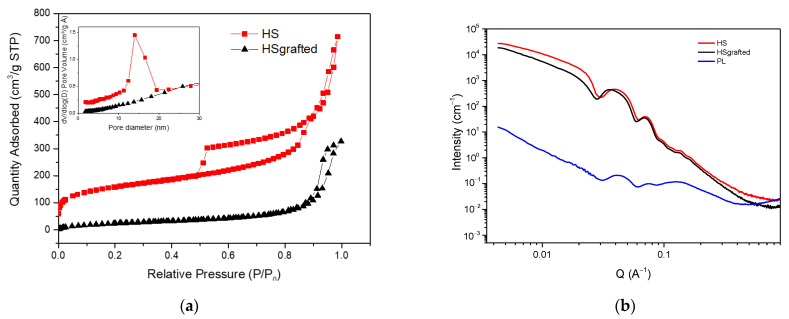
(**a**) N_2_ sorption isotherms of HS and HS grafted, (**b**) Small Angle X-ray Scattering spectra of HS, HS grafted and PL.

The porous and structural properties of these materials were further characterized by N_2_ sorption and SAXS (Figure 4). The sorption results are presented in Figure 4a for the HS and HS grafted samples. The porous liquid could unfortunately not be measured with this technique because N_2_ sorption requires performing measurements at low temperatures (77K) that were observed to damage the liquid sample.

The sorption isotherm of HS is a type IV isotherm according to the IUPAC classification, which confirms that the material presents both micro and mesopores [22,23,24,25]. It is also important to notice that there are two hysteresis loops on this isotherm. The first loop located between 0.5 and 0.9 P/P_0_ is a type H1 loop. It is due to N_2_ sorption in the sample mesopores, being the internal core of the nanospheres. As previously proposed for similar materials [26], the second hysteresis loop located between 0.9 and 1 P/P_0_ was assigned to the capillary condensation of N_2_ molecules between the aggregated nanospheres. The top inset of Figure 4a also shows the pore size distribution derived from the desorption branch of the H1 hysteresis. It confirms the mesopores diameter of ca. 15 nm observed with TEM.

After the organosilane grafting, the disappearance of the first hysteresis loop reveals that the internal cavity of the nanospheres is no more accessible. This is confirmed by the disappearance of the peak of the pore size distribution in the inset of Figure 4a. A specific BET surface has been estimated for these two samples. It shows a significant loss from 491.9 m^2^/g for the HS to 104 m^2^/g for the HS grafted, confirmed at a low P/P_0_ by the disappearance of the microporosity (from 0.09 cm^3^/g to 0). This suggests that the shell is not permeable to N_2_, which may be at the origin of the loss of the mesopores’ accessibility. In the same conditions, Zhang et al. [2] observed a diminution of porosity, with remaining access to the mesoporosity. Concerning the porous liquid, given that it is not possible to conduct N_2_ sorption measurements, a calculation of the supposed porosity has been made, based on TGA results. Considering that the PL is composed of 5 wt% of silica (TGA results), and that the porous volume of HS is 0.53 cm^3^/g (sorption results), it can be estimated that the total porous volume of PL is 0.0265 cm^3^/g, including a microporosity of 0.0045 cm^3^/g. This result is very low compared to the HS sample because the porous liquid is mostly made of organic compounds.

The nanostructure of the samples was further characterized by USAXS and SAXS. In Figure 4b, the spectra show oscillations that are typical of monodispersed spheres [27]. An increase in the intensity at a low Q instead of a plateau suggests the presence of attractive interactions between the nanospheres, which is consistent with the aggregation of nanoparticles observed on the TEM micrographs. HS and HS grafted produce very similar signals, while the porous liquid appears much more attenuated. This might be due to the decrease in the electron density contrast between the silica organic shell for the HS grafted and porous liquid. More importantly, this decrease in the scattering signal can be assigned to the much smaller volume fraction of nanospheres in the porous liquid than in the powder HS samples, as also shown on the TGA results.

A complete fit of these data was performed to provide quantitative information of the nanospheres structure. Thanks to a core-(multi)shell spherical model and a sticky hard sphere structure factor, the core radii, silica and organic shells thicknesses could be estimated. The fitted data and parameters are presented in Supplementary Materials (Figures S1–S3 and Table S1). Considering a microporosity of 6% (estimated from N_2_ isotherms results), an electron density of 16 × 8 × 10^−6^ Å^−1^ was fixed for the silica shell of the HS powder. Consistent with the TEM results, a core diameter of 13.6 nm and a silica shell of 6.5 nm were obtained for this sample.

The HS grafted sample was fitted with the HS powder features as initial parameters. The fit showed that the silica shell is densified after the organosilane grafting, which is consistent with the reduced access to the cavity observed with N_2_ sorption. A better fit was also obtained by considering a low-density solvent around the nanospheres instead of an additional organic shell. This suggests that the nanospheres are aggregated without any void between the organic shells of organosilane.

The LP sample was more complicated to fit because of the presence of a bump between 0.1 and 0.2 Å^−1^. The latter was identified with SANS investigation as hydrophilic domains inside the PEG branches (not detailed here). This suggests that residual hydrophilic species remain present in this sample after the synthesis. Performed on a smaller Q range, the fit of the LP sample shows the presence of a small organic corona of 1 nm consistent with the organosilanes collapsed at the nanospheres external surface. An organic solvent was also found around the nanospheres, which is assigned to the interpenetrated PEG branches. Surprisingly, it appears also that the fit of the PL sample requires considering a very low scaling factor that could not be modelled with a low nanoparticles volume fraction. We interpreted this effect as due to the presence of residual PEG and hydrophilic species located around the aggregated grafted nanospheres.

Additional parameters, such as polydispersity and stickiness parameter, were obtained from the fit. A polydispersity of 0.05 to 0.12 for the core radius and around 0.2 for the shell thickness could be estimated for all the samples. A stickiness parameter was also estimated, showing that the three samples present similar attractive interactions between the nanospheres. The latter simulates the particles aggregation but could also be accounted by a Q^−4^ contribution at a low Q, due to the formation of bigger assemblies of nanospheres.

### 3.2. Effect of Synthesis Parameters

As one of the main interests for the application of these materials is to exploit their empty cavities, this study focuses on the reachability of the core of the nanospheres, by investigating the porosity of the silica shell. All the parameters that may modify the spheres’ structure and porosity have, therefore, been investigated in detail. The calcination temperature, surfactant quantity and the percentage of the oil component have been modified to characterize their effect on the spheres structure and porosity, in order to target optimized properties for the desired applications. As the porous properties of the final PL cannot be measured using N_2_ sorption, we evaluated the effect of the synthesis parameters on the calcinated HS spheres.

#### 3.2.1. Calcination Temperature

After the first synthesis step, a calcination step is required to remove the templating surfactant. As this thermal treatment has a significant impact on the morphology of the silica shell, four calcination temperatures have been tested to evaluate their effect on the nanospheres’ structure, size and porosity.

For this, the HS powders’ microstructure has been followed using SAXS and TEM for calcination temperatures ranging from 450 to 750 °C and compared to the 100 °C dried HS samples.

**Figure 5:**
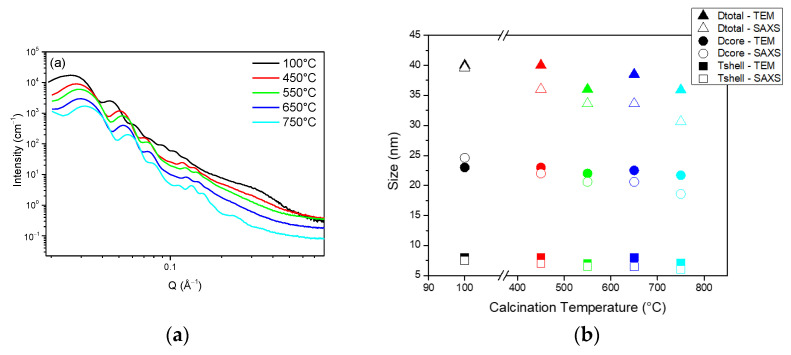
(**a**) SAXS spectra of HS powders for various calcination temperature; (**b**) Corresponding pore diameter and silica shell thickness derived from TEM measurements and SAXS fitting.

Figure 5 shows the SAXS spectra as well as the core sizes and silica shell thicknesses derived from the SAXS fitting and TEM images. As in Figure 4b, the SAXS spectra show oscillations that are typical of monodispersed spheres. These oscillations are shifted to the higher Q range for the higher calcination temperatures, which indicates that the size of the spheres is decreasing. A complete fit of the SAXS data allowed the temperature effect on the core size and shell thickness to be evaluated (See fitting parameters in Table S2). The results are plotted in Figure 5b and compared to the sizes derived from the TEM images. Both techniques show that both the core sizes and shell thicknesses are impacted by the calcination. A 10% decrease from 100 to 750 °C is attributed to the densification of the silica matrix.

To confirm this trend, N_2_ sorption was applied to evaluate the effect of calcination temperature on the spheres’ porosity (See sorption isotherm in Figure S1 and derived parameters in Table 1). As expected, a decrease in the total porous volume shows that the silica material is densified with the calcination temperature. Consistent with the shell contraction observed with the TEM, the microporosity of the material is also decreased by a factor of 10. At 750 °C, the hysteresis is not visible anymore, indicating that the cores are no longer accessible and that the microporosity has completely disappeared.

The BET surface areas of 582.4 to 89.5 m^2^/g for calcination temperatures of 450 to 750 °C confirm the loss of porosity. The microporous volumes also confirm a silica densification, with values going from 0.14 cm^3^/g at 450 °C to a negligible value of 0.01 cm^3^/g at 750 °C.

These results indicate that for the highest temperatures, the silica network is contracted until the internal cavities of the spheres are no longer accessible because of a loss of microporosity.

Overall, this study shows that the calcination temperature affects the core and the shell sizes, with a global densification of the silica shell. An increase in the temperature reduces the microporosity of the shell, leading to limited access to the internal cavity of the spheres. As the TGA results show that no organic compounds remain present after 400 °C, it can be considered that an optimal temperature of 450 °C is necessary to achieve a proper calcination while maintaining a high porosity in the silica shell and the access to the internal cavities.

#### 3.2.2. Effect of Surfactant Concentration

Since the surfactant (here the triblock copolymer F127) may form monomeric to spherical or cylindrical micelles in its phase diagram [28], it is expected to influence the structure and the shape of the resultant nanoparticles.

**Figure 6:**
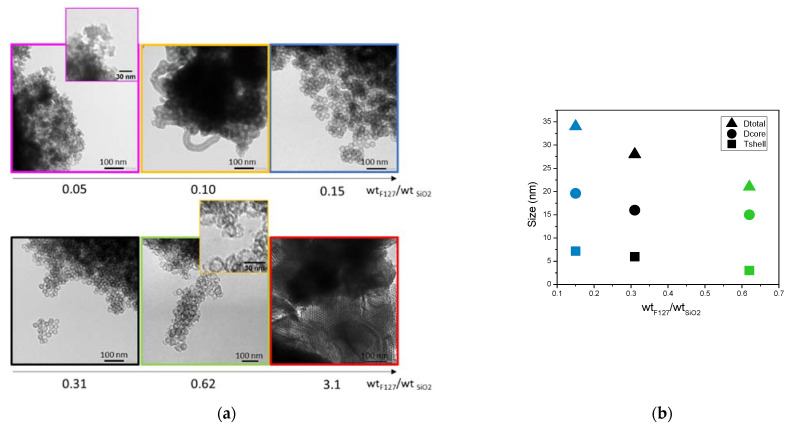
(**a**) TEM images and (**b**) corresponding size measurements of nanospheres as a function F127/silica weight ratio.

Six surfactant-to-silica weight ratios have, therefore, been tested. Figure 6 shows the TEM micrographs and the derived size parameters of the spheres.

As observed on the TEM micrographs, hollow spheres are formed for the surfactant-to-silica ratios between 0.10 and 0.62. Below and over these two limits, no spheres are visible. For a ratio of 0.62, it can be noticed that the spheres present thinner silica shells that appear broken in some cases. Since cylindrical particles are also formed, 0.10 of F127/SiO_2_ is also a limit case. In the following, only the ratios 0.15, 0.31 and 0.62 presenting well shaped hollow nanospheres were characterized in more detail. The size evaluation in Figure 6b shows that not only the size of the core, but also the thickness of the shell is decreased when the ratio of surfactant to silica is increased. However, these decreases do not occur in the same proportions. The decrease in the shell thickness becomes significant and reaches a collapse of 60% at a ratio of 0.62 of F127, while the core size is only reduced by 15%.

Table 2 details the values obtained from N_2_ sorption for these three ratios.

**Figure 7:**
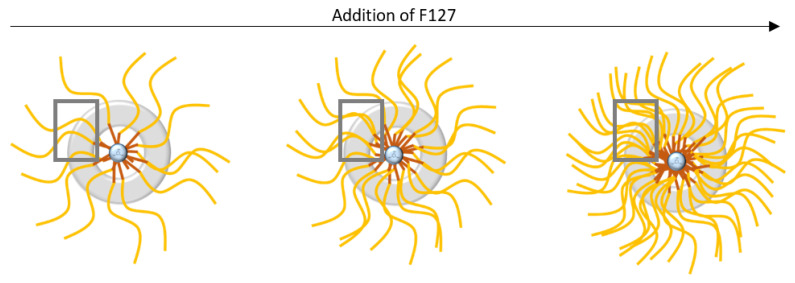
Schematic illustration of the influence of F127 addition during the synthesis of the nanospheres.

The first observation is that the BET surface areas and total porous volume increase significantly when more F127 is added. This can be attributed both to the increase in the number of F127 formed micelles, leading to more silica nanospheres, and to a higher microporosity in the silica shell of the nanospheres. Indeed, as proposed by Managa and Kerkhoff [29,30,31] and as illustrated in Figure 7, the silica network polycondenses between the surfactant’s F127 hydrophilic branches. After the surfactant’s removal by calcination, the footprint of the hydrophilic branches forms micropores in the silica shell, while the hydrophobic ones (together, with the oily swelling agent (TMB)) form the internal cavity of the spheres (mesopores). Consequently, the higher the number of present hydrophilic branches of F127 is, the more microporous the material is. Considering the effect of the surfactant concentration on the sphere formation mechanism illustrated in Figure 7, it can also be estimated that in the 0.62 wt% case (higher limit of surfactant content), the numerous branches of surfactant do not allow the silica to polycondense properly, leading to thinner and brittle silica corona, as observed on the TEM micrograph.

The surfactant addition seems, therefore, to markedly affect the morphology of the silica shell. The microporosity created by the F127 branches is increased for a higher F127 content, with reduced sizes of the silica shell. This study shows, therefore, that the silica to surfactant ratio has to be chosen between 0.10 and 0.62 to obtain well shaped hollow nanospheres. In this range of ratio, we show, moreover, that a higher F127 content favors not only bigger sizes of the internal cavities, but also a better accessibility to these cavities thanks to higher silica microporosities.

#### 3.2.3. Effect of Trimethylbenzene Addition

As illustrated above, the surfactant content may significantly influence not only the core size of the nanospheres, but also the microporous properties of the hollow nanospheres. The last essential parameter investigated in this study is the amount of trimethylbenzene (TMB). TMB is conventionally added as a fatty swelling agent to increase the hydrophobic cores of direct micelles and to increase the pore size of mesoporous materials [32,33,34,35]. In their synthesis procedure, Zhang et al. proposed adding a TMB/Silica weight ratio of 0.31 without justifying either its requirement or its impact. The synthesis was here reproduced with TMB/Silica weight ratios of 0, 0.31 and 0.54 to evaluate its influence on the hollow spheres’ nanostructure and porous properties.

**Figure 8:**
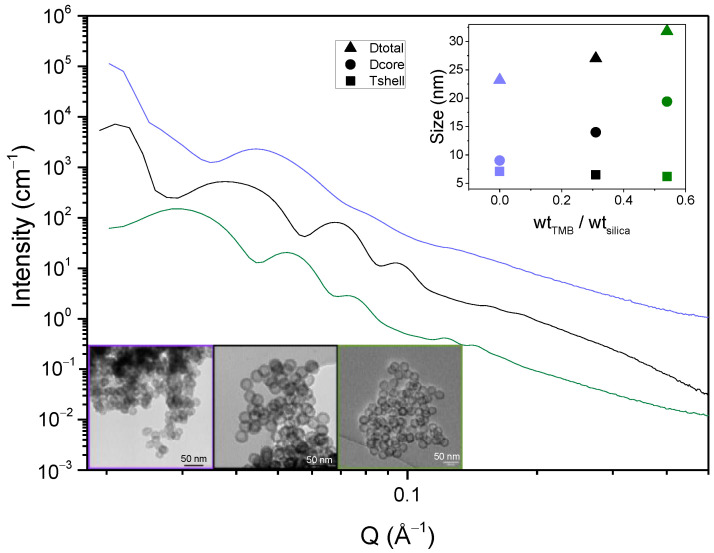
SAXS spectra of HS powders for 0, 0.31 and 0.54 TMB/silica weight ratios; insets: TEM micrographs and size measurements of the spheres.

Figure 8 presents the SAXS spectra, the TEM micrographs and the derived size parameters for these three TMB/Silica weight ratios. For a ratio of 0, TEM shows that the cores of the spheres are not well defined, while they are well shaped for the two other ratios. As shown in the top inset of Figure 8, the core size is significantly increased by the TMB addition, while the silica shell size is slightly decreased. In the absence of TMB, the SAXS spectra show less defined oscillations, suggesting that the spheres are less monodispersed and well designed. This observation is also confirmed by the TEM images.

**Figure 9:**
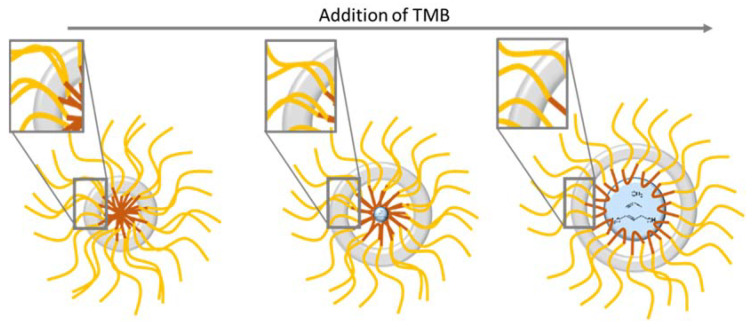
Schematic illustration of the influence of TMB addition in the synthesis of the nanospheres.

The SAXS oscillations are also clearly shifted to the smaller Q range for the higher TMB content, which confirms that the spheres are swollen and bigger when TMB is added. This study justifies the need of TMB in the Zhang at al. protocol, as a small amount of TMB is required to form well defined spherical internal cavities. As illustrated in Figure 9, TMB is indeed swelling the hydrophobic core of the F127 micelles, inducing the formation of hollow spheres with bigger internal cavities.

To evaluate the effect of TMB on the silica spheres porosity, the N_2_ sorption isotherms were measured on the same three samples. The isotherms are presented in Figure S3 and the derived porous volumes and BET surface areas are given in Table 3.

The porous properties show an optimum for the TMB ratio of 0.31. At this ratio, the obtained material presents the higher microporosity and total porosity as well as the higher BET surface area.

To interpret this surprising result, the hollow sphere formation mechanism illustrated in Figure 9 has to be taken into account. As mentioned before, the silica shells are templated by the hydrophobic branches of the F127 surfactants molecules. For a constant F127 amount, the increase in TMB is expected to induce a decrease in the density of the F127 hydrophobic branches in the silica shells (See illustration in the square zoom of Figure 9). This explains the decrease in microporosity between 0.31 and 0.54 of TMB/SiO_2_. However, according to this hypothesis, the complete absence of TMB should lead to a very dense quantity of hydrophobic branches expected to produce an even higher microporosity in the silica shell. To interpret the apparent contradiction, it has to be considered that the silica shell is also shown to be thicker for the lower content of TMB. The decrease in the microporosity might then be due to the slightly less connected micropores in these thicker silica shells.

It is confirmed here that TMB is a swelling agent of surfactant micelles, affecting, in the present case, the core size of the hollow nanospheres. We observe, moreover, that the amount of TMB has a significant influence not only on the silica shell microporosity but also on its thickness. This secondary and unexpected effect shows that the TMB content has to be finely tuned for further applications to target both the optimized size of internal cavities and a good accessibility to these cavities.

## 4. Conclusions

In this article, the different steps of a type I porous liquid synthesis were characterized in detail. Based on surfactant templated silica nanoparticles, silica hollow spheres, as well as grafted spheres with organosilane and a PEG canopy were shown to provide porous liquids with very monodispersed cavities. The efficiency of the different synthesis steps was also confirmed with TGA and FTIR, giving quantitative information of the composition of the final samples. The accessibility of the internal core of the nanospheres was investigated with N_2_ sorption isotherms. While such a gas is not representative of the sphere permeability to any fluids, it was shown that the grafting of organic functions at the surface of the sphere drastically affected the N_2_ accessibility to the silica micropores and, consequently, to the internal mesopores.

In order to understand the synthesis mechanism of this type of porous liquid and to help the optimization of the resulting materials for applications, the second part of this study investigated the effect of the main synthesis parameters on the hollow nanospheres’ structure and porous properties. It was found that the calcination temperature applied to remove the templating surfactant induces a decrease in the core and silica shell sizes associated to a decrease in silica microporosity. The surfactant amount was also varied. The study shows that a range of surfactant-to-silica ratios have to be respected to form well defined spherical nanospheres. This parameter also has a strong influence on the silica microporosity, which could be explained by the increased number of hydrophilic surfactant branches incorporated in the silica shell during the synthesis. Higher surfactant contents are, therefore, favorable to obtain better accessibility to the internal cavities. Finally, the addition of TMB in the synthesis was shown to be a requisite to form well shaped hollow nanospheres. It was confirmed that this hydrophobic swelling agent of micelles increases the core size of the nanospheres. Less expectedly, it was also shown to significantly affect the silica shell microporosity with a nonlinear effect; low amounts of TMB induce low microporosity attributed to a thicker silica shell and less connected micropores. Increasing the amount of TMB tends to reach a maximum of microporosity, which is, again, decreased when the shell curvature increase induces a decrease in the surfactant hydrophilic branches in the silica.

This study gives key parameters to design the well-defined and permeable hollow nanospheres necessary to form porous liquid that are potentially interesting for applications. We show that the synthesis step can be tuned to adapt the porosity of the silica shell, the accessibility and the size of their internal cavities or to extend the global size of the spheres. It is important to notice that the important properties of the final material can be controlled from the first steps of hollow spheres synthesis, by understanding their formation mechanisms. Poor accessibility to gas was observed for the materials grafted with an organic function, which suggests that the final porous liquid is poorly accessible to N_2_. This observation questions the accessibility of porous liquid porosity to any gas or liquid, as the organic grafting might clog the microporosity. In order to consider porous liquids for broader applications, as, for example, in separative chemistry, further investigations would require evaluating the accessibility of these materials to solvents that may be exploited to transport solutes to extract.

## Figures and Tables

**Table 1:** BET surface area, microporous and total porous volumes obtained from N_2_ sorption as a function of the calcination temperature.

Temperature (°C)	450	550	650	750
S_BET_ (cm^2^/g STP)	582.4	268.0	149.6	89.5
V_µporous_ (cm^3^/g)	0.14	0.05	0.02	0.01
V_total_ (cm^3^/g)	0.56	0.34	0.21	0.12

**Table 2:** Specific areas and porous volumes of HS powders as a function of F127/silica weight ratio.

Wt_F127_/Wt_SiO2_	0.15	0.31	0.62
S_BET_ (cm^2^/g STP)	361.7	491.9	702.3
V_µporous_ (cm^3^/g)	0.06	0.09	0.11
V_total_ (cm^3^/g)	0.4	0.53	1.2

**Table 3:** Specific areas and porous volumes of HS powders as a function of TMB/silica weight ratio.

Wt_TMB_/Wt_SiO2_	0	0.31	0.54
S_BET_ (cm^2^/g STP)	382.2	491.9	268.0
V_µporous_ (cm^3^/g)	0.06	0.09	0.05
V_total_ (cm^3^/g)	0.35	0.53	0.34

## Supplementary Materials

The following are available online at https://www.mdpi.com/article/10.3390/nano11092307/s1, Figure S1: SAXS spectra (experiment and fit) of hollow spheres according to the parameters in Table S1, Figure S2: SAXS spectra (experiment and fit) of hollow grafted spheres according to the parameters in Table S1, Figure S3: SAXS spectra (experiment and fit) of porous liquid according to the parameters in Table S1, Figure S4: N_2_ sorption isotherms of HS powders for various calcination temperatures, Figure S5: N_2_ sorption isotherms of HS powders for 0.15, 0.31 and 0.62% of F127/silica weight ratios, Figure S6: N_2_ sorption isotherms of HS powders for 0, 0.31 and 0.54% of TMB/silica weight ratios, Table S1: SAXS fitting parameters of HS, HS grafted and PL samples, Table S2: SAXS fitting parameters of HS samples for various calcination temperatures.
