# Supplementary material for: Key Parameters to Tailor Hollow Silica Nanospheres for a Type I Porous Liquid Synthesis: Optimized Structure and Accessibility
Source: Nanomaterials (Basel). 2021 Sep 6;11(9):2307. doi: 10.3390/nano11092307 (PMC8465660; doi:10.3390/nano11092307)
Supplement: Supplementary file 1 [file nanomaterials-11-02307-s001.zip › nanomaterials-1338503-supplementary.pdf]

Supplementary informations

Key Parameters to Tailor Hollow Silica Nanospheres for a Type I Porous Liquid Synthesis: Optimized Structure and Accessibility

Justine Ben Ghozi-Bouvrande, Stéphane Pellet-Rostaing and Sandrine Dourdain *

ICSM, Univ Montpellier, CEA, CNRS, ENSCM, 30207Marcoule, France; justine.benghozibouvrande@cea.fr (J.B.G.-B.); stephane.pellet-rostaing@cea.fr (S.P.-R.)

* Correspondence: sandrine.dourdain@cea.fr

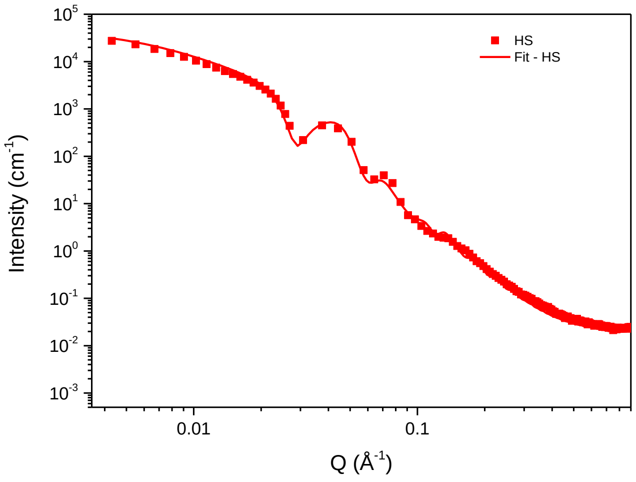

Figure S1. SAXS spectra (experiment and fit) of hollow spheresaccording to the parameters in **Error! Reference source not found.**.

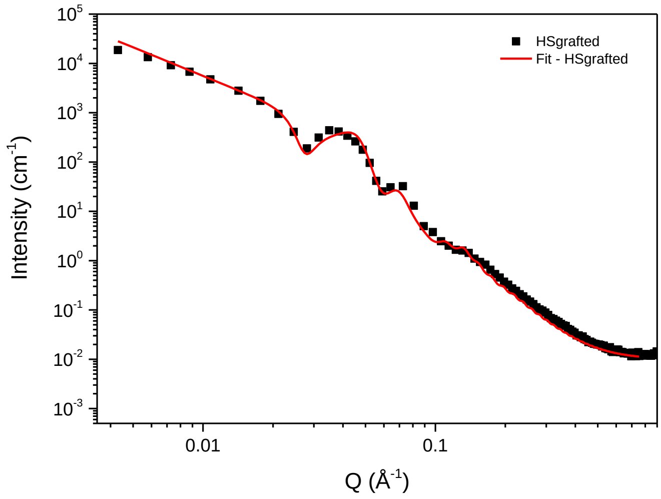

Figure S2. SAXS spectra (experiment and fit) of hollow grafted spheres according to the parameters in **Error! Reference source not found.**.

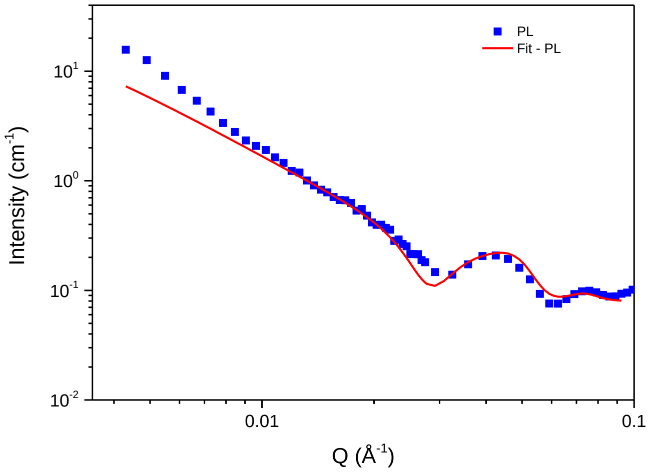

Figure S3. SAXS spectra (experiment and fit) of porous liquid according to the parameters in **Error! Reference source not found.**.

Table S1. SAXS fitting parameters of HS, HS grafted and PL samples.

Sample (°C)	Form factor Core-Shell Sphere						
	Background	Scale	Radius	sld_core	sld_solvent	shell	sld_shell
100	0.01	0.25	123	0	0	75	19
450	0.05	0.6	110	0	0	70	19
550	0.08	0.7	103	0	0	65	19
650	0.08	0.7	103	0	0	65	19
750	0.09	0.7	98	0	0	60	19

Sample	Polydispersity		Structure factor Sticky Hard Sphere		
	PD radius	PD thickness 1	Vol Fraction	Perturb	Stickiness
100	0.07	0.3	0.2	0.05	0.2
450	0.05	0.2	0.2	0.05	0.2
550	0.05	0.2	0.2	0.05	0.2
650	0.05	0.2	0.2	0.05	0.2
750	0.05	0.15	0.2	0.05	0.2

sld	10^{-6}/A^2
shell	A

Table S2. SAXS fitting parameters of HS sample for various calcination temperatures.

Sample	Form factor Core-(multi)Shell Sphere								
	Background	Scale	Radius	sld_core	sld_solvent	shell 1	sld_shell 1	shell 2	sld_shell 2
HS	0.02	1	68	0	0	65	16.8	/	/
HS Grafted	0.01	1	68	0	5	65	18	/	/
PL	0.08	0.001	67	0	7	60	18	10	5

Sample	Polydispersity			Structure factor Sticky Hard Sphere		
	PD radius	PD thickness 1	PD thickness 2	Vol Fraction	Perturb	Stickiness
HS	0.05	0.25	/	0.12	0.09	0.16
HS Grafted	0.12	0.2	/	0.12	0.09	0.1
PL	0.12	0	0	0.07	0	0.092

sld	10^{-6}/A^2
shell	A

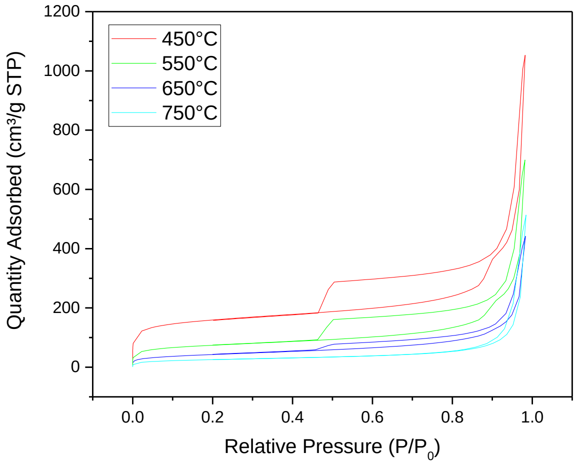

Figure S4. N_2 sorption isotherms of HS powders for various calcination temperatures.

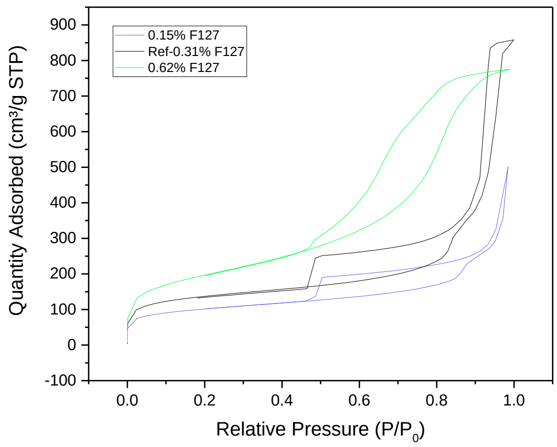

Figure S5. N_2 sorption isotherms of HS powders for 0.15, 0.31 and 0.62% of F127/silica weight ratios.

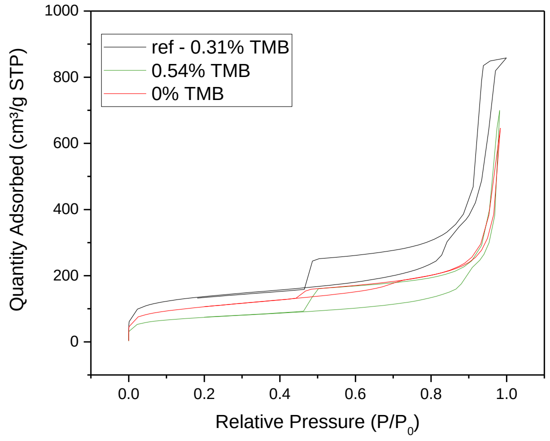

Figure S6. N_2 sorption isotherm of HS powders for 0, 0.31 and 0.54 % of TMB/silica weight ratios.
